# Comparison of Channel Catfish and Blue Catfish Gut Microbiota Assemblages Shows Minimal Effects of Host Genetics on Microbial Structure and Inferred Function

**DOI:** 10.3389/fmicb.2018.01073

**Published:** 2018-05-23

**Authors:** Jacob W. Bledsoe, Geoffrey C. Waldbieser, Kelly S. Swanson, Brian C. Peterson, Brian C. Small

**Affiliations:** ^1^Aquaculture Research Institute, University of Idaho, Hagerman, ID, United States; ^2^Warmwater Aquaculture Research Unit, US Department of Agriculture – Agriculture Research Services, Stoneville, MS, United States; ^3^Department of Animal Science, University of Illinois at Urbana–Champaign, Urbana, IL, United States; ^4^National Cold Water Marine Aquaculture Center, US Department of Agriculture – Agriculture Research Services, Franklin, ME, United States

**Keywords:** fish microbiome, *Ictaluridae*, aquaculture microbiology, environmental microbiota, channel catfish, blue catfish

## Abstract

The microbiota of teleost fish has gained a great deal of research attention within the past decade, with experiments suggesting that both host-genetics and environment are strong ecological forces shaping the bacterial assemblages of fish microbiomes. Despite representing great commercial and scientific importance, the catfish within the family *Ictaluridae*, specifically the blue and channel catfish, have received very little research attention directed toward their gut-associated microbiota using 16S rRNA gene sequencing. Within this study we utilize multiple genetically distinct strains of blue and channel catfish, verified via microsatellite genotyping, to further quantify the role of host-genetics in shaping the bacterial communities in the fish gut, while maintaining environmental and husbandry parameters constant. Comparisons of the gut microbiota among the two catfish species showed no differences in bacterial species richness (observed and Chao1) or overall composition (weighted and unweighted UniFrac) and UniFrac distances showed no correlation with host genetic distances (Rst) according to Mantel tests. The microbiota of environmental samples (diet and water) were found to be significantly more diverse than that of the catfish gut associated samples, suggesting that factors within the host were further regulating the bacterial communities, despite the lack of a clear connection between microbiota composition and host genotype. The catfish gut communities were dominated by the phyla Fusobacteria, Proteobacteria, and Firmicutes; however, differential abundance analysis between the two catfish species using analysis of composition of microbiomes detected two differential genera, *Cetobacterium* and *Clostridium XI*. The metagenomic pathway features inferred from our dataset suggests the catfish gut bacterial communities possess pathways beneficial to their host such as those involved in nutrient metabolism and antimicrobial biosynthesis, while also containing pathways involved in virulence factors of pathogens. Testing of the inferred KEGG (Kyoto Encyclopedia of Genes and Genomes) pathways by DESeq2 revealed minor difference in microbiota function, with only two metagenomic pathways detected as differentially abundant between the two catfish species. As the first study to characterize the gut microbiota of blue catfish, our study results have direct implications on future ictalurid catfish research. Additionally, our insight into the intrinsic factors driving microbiota structure has basic implications for the future study of fish gut microbiota.

## Introduction

Recently the aquaculture industry has seen a great deal of interest in pre-, pro-, and synbiotic supplementation to modulate the gut microbiota of fish, primarily due to their anticipated efficacy in improving growth rates, feed conversion ratios, and replacing the use of antibiotics to maintain fish health. This interest has in turn stimulated much research on the microbiome of cultured teleost fish species. Over the past decade, the physiological utility of the gut-associated microbiota of teleosts has been proven, with many studies implicating the microbiota in host functions such as lipid metabolism and absorption (Semova et al., 2012), vitamin and mineral metabolism (Tsuchiya et al., 2008), digestion of complex feedstuffs (Wu et al., 2012; Di Maiuta et al., 2013; Ni et al., 2014), disease resistance and immune function (Pérez et al., 2010; Galindo-Villegas et al., 2012; Ran et al., 2012; Gomez et al., 2013), and development of the gastrointestinal tract and other tissues (Rawls et al., 2004; Hill et al., 2016). While this body of research has indicated that the microbiota of fish certainly merits research attention due to their known function, the challenge lies in controlling the variability of the teleost microbiota and better understanding the factors affecting microbial community assemblages in cultured fish species to allow for more effective manipulation of their composition and function. Previous studies have shown that the teleost gut microbiota varies according to many host-associated factors, including ontogenetic development (Stephens et al., 2015; Bledsoe et al., 2016), diet (Zhou et al., 2013; Ingerslev et al., 2014; Wong et al., 2015), environment (Sullam et al., 2012; Wong and Rawls, 2012; Giatsis et al., 2014; Dehler et al., 2017), as well as host genetics and phylogeny (Li X. et al., 2012; Navarrete et al., 2012; Sullam et al., 2012; Li J. et al., 2014; Smith et al., 2015).

Of these variables known to shape the gut-associated microbiota of fish, host genetics is of particular interest. If a strong correlation among fish genotype and a desirable gut microbial community with beneficial function can be established, it would suggest that the host traits are heritable. Therefore, a superior performing microbiota could be achieved through selection within aquaculture breeding programs (Llewellyn et al., 2014). To date, the effects of host genetics on the structure and function of the teleost gut microbiota are still somewhat unclear, although some studies suggest that differences in host genotype can result in clear distinctions in gut microbiota composition. In a study using gel-electrophoresis techniques to compare the gut microbiota of four families of rainbow trout *Oncorhynchus mykiss* fed three different diets, Navarrete et al. (2012) observed that host family, not dietary treatment, best explained differences in the gut microbiota composition. In another study also using gel-based techniques, Li X. et al. (2012) observed that the larvae of four different species within the family Cyprinidae possessed distinct gut bacterial communities even when cohabitated. These results suggest that host genotype serves as a strong determinant in shaping the gut bacterial communities of aquacultured teleost species. However, more research on this topic using next-generation sequencing methods is needed, especially when considering the large variation in fish species and husbandry techniques utilized across the aquaculture industry.

The commercially important catfish within the family *Ictaluridae*, particularly the channel catfish *Ictalurus punctatus* and blue catfish *I. furcatus*, represent an ideal system for further evaluating this relationship between host genetics and the gut-associated microbiota. Channel catfish currently represent the greatest market share of the US freshwater aquaculture industry (approximately 65%) (Hanson and Sites, 2015), with an estimated commercial food fish value of just under 386 million dollars within the United States (USDA NASS, 2016). Additionally, a congener, the blue catfish, is also of great interest to the commercial aquaculture industry because interspecific crosses (*I. punctatus* ♀ × *I. furcatus* ♂) produce offspring with desirable phenotypes including superior disease resistance and fillet yield (Li et al., 2004). In addition to the commercial importance of these two species, a wealth of literature exists regarding their genetics (Liu et al., 1998, 2016; Quiniou et al., 2007; Wang et al., 2010), physiology (Li et al., 2004; Small, 2006a; Stewart and Allen, 2014), and mucosal immunity (Peterson et al., 2005; Li C. et al., 2012, 2014; Zhang et al., 2012). Many of these studies show the two congeners to exhibit marked differences in physiologic indices which are of great relevance to the gut-associated microbiota. Despite this rationale, only two studies have explored the gut microbiota of channel catfish using modern sequencing methods, and no studies have been published relating to the gut microbiota of blue catfish. The first study on the gut microbiota of channel catfish characterized the gut microbiota of individuals (*n* = 5) collected from a recreational fishing pond (Larsen et al., 2014). In the only other microbiota study on ictalurid catfish, our group tracked the temporal changes of the channel catfish microbiota across the first 193 days of life (Bledsoe et al., 2016). Together, these previous studies have shown catfish gut microbiota to be relatively simple communities, dominated by microbes from the phyla Fusobacteria and Proteobacteria. Additionally, the catfish gut microbiota appears to be rather dynamic in early life stages, yet, variability in microbiota composition seems to diminish later in life toward 193 days post hatch (dph) (Bledsoe et al., 2016). More research is needed to fully understand the gut microbiota of these valuable aquaculture species, and no studies have explored the effect of host genetics on shaping these microbial communities.

As such, the primary aim of the present research was to characterize and compare the gut microbiota of genetically distinct strains of channel catfish and blue catfish to determine if differences in host-genotype also lead to differences in gut microbiota structure and function when all environmental variables are held constant. To evaluate the effects of host genetics on the gut microbiota of these species, three strains of *I. punctatus* (USDA103, USDA503, and Delta Select) and three strains of *I. furcatus* (D&B, Mississippi River, and Rio Grande River) were selected for inclusion in this study. The USDA103 strain is a channel catfish research strain known for high-feed intake that was developed by the US Department of Agriculture – Agriculture Research Services (USDA-ARS) through selection for growth performance (Wolters et al., 2000; Small, 2006a). The USDA503 strain, also a channel catfish research strain developed by the USDA-ARS, was derived from USDA103 individuals with continued selection pressures placed on growth performance. Delta Select is a commercial strain of channel catfish developed and maintained by the USDA-ARS Warmwater Aquaculture Research Unit (WARU), originating from individuals collected from 10 commercial facilities located in the epicenter of US commercial ictalurid production, the Mississippi Delta (Stewart and Allen, 2014). The D&B blue catfish strain originated from the Arkansas and Mississippi River, before undergoing commercial selection through crosses with other commercial strains (Dunham and Smitherman, 1984). The Rio Grande and the Mississippi River strain of blue catfish used in this study are domesticated, yet unselected, strains of blue catfish that originated from random mating of wild fish collected from the Rio Grande and Mississippi River, respectively (Dunham and Smitherman, 1984).

## Materials and Methods

All animal experiments were conducted at the USDA-ARS WARU of the National Warmwater Aquaculture Center (NWAC), Stoneville, MS, United States, in accordance with the experimental research protocol approved by the Institutional Animal Care and Use Committee (64-F-006-6803).

### Study Design and Sampling

**FIGURE 1:**
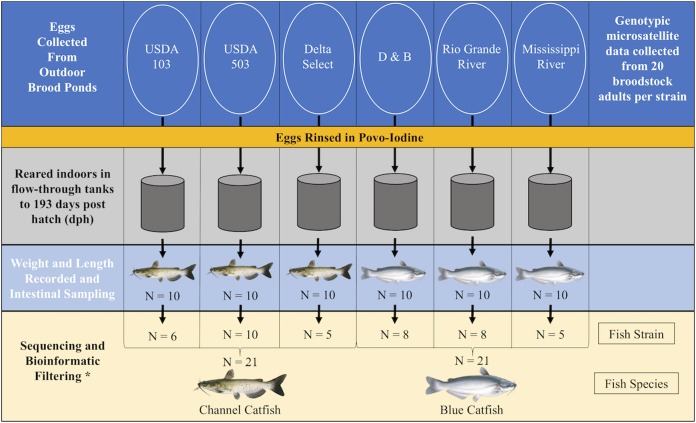
Schematic outlining the design of the study and the sample size throughout each step. Samples were required to contain 10,000 classified SVs for inclusion in the downstream analysis, explaining the reduction in sample size at the bioinformatic filtering step (^∗^). Images © Joseph R. Tomelleri.

In early July, eggs from multiple families within each of the selected strains were collected from outdoor spawning ponds at NWAC and rinsed in a 100 ppm povidone-iodine solution at the time of collection as per industry standard practices (Small, 2006b). Eggs were hatched indoors in flow-through raceways and upon hatching the multi-family cohorts within each strain were transferred to individual 76 L rearing tanks (one tank per strain) in a common system supplied with constant flow-through well water (3.8 L min^-1^, ∼26°C, pH ∼8.5, dissolved oxygen >5 ppm) (**Figure 1**). Fish densities within each tank were maintained relatively equal over time through random culling; however, no size selective grading was conducted. All fish were fed to apparent satiation daily using the same commercially available diet (Supplementary Table S1). At 193 dph, 10 fish were sampled from each strain (**Figure 1**). Fish were euthanized in a solution of 300 mg L^-1^ tricaine methanesulfonate (MS-222; Western Chemical, Ferndale, WA, United States) buffered with sodium bicarbonate in water taken from the culture tanks. Fish length and weight were recorded prior to rinsing the ventral surface of the fish with 75% ethanol, then excising the entire intestinal tract and its contents using sterile procedures. Intestinal samples were immediately flash frozen, transported to the laboratory and stored at -80°C until further processing.

### DNA Extraction and Sequencing

Frozen intestinal samples were homogenized to a powder using individual sterilized mortar and pestles partially submerged in liquid nitrogen. The PowerFecal^®^ DNA Isolation Kit (MoBio Laboratories, Carlsbad, CA, United States) was then used to isolate DNA following the manufacturer’s recommendations. DNA was checked for quality and concentration using a NanoDrop 2000c (ThermoFisher, Wilmington, DE, United States) and gel electrophoresis. DNA samples were then submitted to the Roy J. Carver Biotechnology Center (University of Illinois, Champaign, IL, United States) for preparation and sequencing of 16S rRNA V4 gene amplicons. Sequencing libraries were prepared using 505f and 806r target-specific PCR primers (Caporaso et al., 2011), CS1 and CS2 spacer pads, sample-specific 10 nucleotide (nt) barcodes, and sequencing adapters. Libraries were then sequenced on an Illumina MiSeq platform (Illumina, San Diego, CA, United States). Additionally, microbiota data for one diet-associated and two water-associated samples were obtained from an overlapping study (Bledsoe et al., 2016), corresponding to the diet and the water supplied to rearing tanks at the time of sampling.

### Data Availability

Raw sequence reads from this study are publicly available in the NCBI SRA depository within BioProject PRJNA329560. Fish gut-associated microbiota sequences are available under BioSample accession numbers SAMN07830122–SAMN07830163. Sequence reads from the two water microbiota samples and single diet microbiota sample used in this study are available under the accession numbers SAMN05420564, SAMN05420565, and SAMN05420571, respectively.

### Bioinformatics and Inferred Microbial Function

Raw demultiplexed sequences were processed in R (R Core Team, 2013) using the dada2 package (Callahan et al., 2016) within Bioconductor (Gentleman et al., 2004) by first trimming PCR primers and merging the paired-end sequences after truncating forward and reverse reads at 245 and 160 nt, respectively, to ensure the highest quality of the merged 16S rRNA V4 amplicons (approx. 252 nt). In addition, the merged sequences were quality filtered at a threshold of two expected errors (Edgar and Flyvbjerg, 2015) and no ambiguous nucleotide reads were allowed. The dada2 algorithm was used to infer error-corrected sequence variants (SVs), a classification similar to the typical OTU, yet more accurate, as the Poisson-based error probability model learned from the dataset by dada2 infers exact SVs without clustering multiple unique sequences into single OTUs based on 97% sequence identity. Putative chimeric sequences were removed, and taxonomy was applied to SVs using the RDP v14 reference dataset and naïve Bayesian classifier (Wang et al., 2007; Cole et al., 2014). A phylogenetic tree was assembled using the packages decipher (Wright, 2016) and phangorn (Schliep, 2010) for use in phylogenetically informed beta-diversity metrics. The phyloseq package (McMurdie and Holmes, 2013) was then used for all downstream analyses. SVs assigned to cyanobacteria/chloroplast, NA, or unknown at the phylum level were removed prior to further analysis. Due to the presence of some poorly sequenced sample libraries, samples with less than 10,000 assigned SVs were filtered from the dataset. Sample filtering resulted in even sample sizes at the level of fish species (*I. punctatus n* = 21; *I. furcatus n* = 21), yet the resulting sample sizes were uneven at the level of fish strain (**Figure 1**).

To examine the possible function of the microbiota detected in the gut of these catfish and determine whether microbiota functions differed among host genetics, piphillin was used to normalize our amplicon data by 16S rRNA gene copy number and infer metagenomic contents (Iwai et al., 2016). Following bioinformatic processing, a raw SV-count-table and the associated representative sequences were submitted to piphillin. For the analysis, a sequence identity cut-off of 97% was implemented, and the inferred metagenomic functions were assigned using the Kyoto Encyclopedia of Genes and Genomes database (KEGG; May 2017 Release).

### Host Microsatellite Genotyping

In addition to microbiota data, host genetic data were gathered separately from brood stock adults (*n* = 20) from each strain (**Figure 1**). Host genetic data, in the form of microsatellite genotypes, were generated by isolating DNA from whole blood samples and amplifying 22 known microsatellite loci using locus-specific primers (Supplementary Table S2) and sample-specific barcodes, prior to sequencing on the Illumina MiSeq (Waldbieser and Bosworth, 2013). The resulting dataset was demultiplexed by individual and a custom script was used to identify locus-specific reads and determine the length of the tandem repeat regions (Supplementary Table S2) to resolve allele length at each loci. GENEPOP 4.5.1 (Raymond, 1995; Cock et al., 2009) was used to assess observed and expected heterozygosity, while GenAlEx 6.5 (Peakall and Smouse, 2012) was used to perform an analysis of molecular variation (AMOVA) with Rst genetic distance estimations (Slatkin, 1995).

### Statistical Analyses

All statistical analyses were conducted using packages within R, with a significance threshold of *P* ≤ 0.05. Fish weights were tested for homoscedasticity (Bartlett test) and normality (Shapiro–Wilk test) followed by a nested one-way ANOVA (fish strain nested within fish species). Microbiota alpha-diversity indices (observed and Chao1) among the gut-associated fish samples were also analyzed by nested one-way ANOVA after testing assumptions, which led to a log transformation of Chao1 data. Additionally, a Wilcoxon rank-sum test was conducted to test the alpha-diversity between all gut-associated samples and the environment-associated samples (diet and water). To account for differences in library size, differential abundance analysis of the detected microbiota was conducted on a proportional (relative abundance) taxonomy table agglomerated at the genus level using analysis of composition of microbiomes (ANCOM) (Mandal et al., 2015). ANCOM was conducted after removing spurious observations (SVs with relative abundance below 1e^-5^), using default parameters and a FDR-corrected significance threshold of 0.05. To test whether the potential function of the gut microbiota differed between the two catfish species, the raw KEGG pathway output from piphillin was analyzed by DESeq2 using default parameters, after flooring fractional counts to the nearest integer (Love et al., 2014). The inferred metagenomic pathways were considered differentially abundant using a FDR-corrected significance threshold of 0.05. Microbiota beta-diversity was assessed using weighted and unweighted UniFrac after rarefying the data, without replacement, to the minimum library size (11,631 SVs) to ensure results were not biased by sampling depth (Weiss et al., 2017). The package vegan (Oksanen et al., 2017) was used to conduct a permutational analysis of multivariate dispersion (PERMDISP2) (Anderson, 2006) at the level of fish species and to test for overall differences in beta-diversity, a nested (fish strain within fish species) permutational multivariate analysis of variance (PERMANOVA) was conducted on both UniFrac metrics (BiodiversityR; Kindt and Coe, 2008). To test for correlations among host genetics and gut microbiota composition, a Mantel test was conducted on Pearson correlation coefficients between strain-wise microsatellite-based host genetic distances (Rst) and mean strain-wise microbiota beta-diversity distances, with permutations stratified by fish species. All permutational tests were conducted using 999 permutations unless otherwise dictated by data structure.

## Results

**FIGURE 2:**
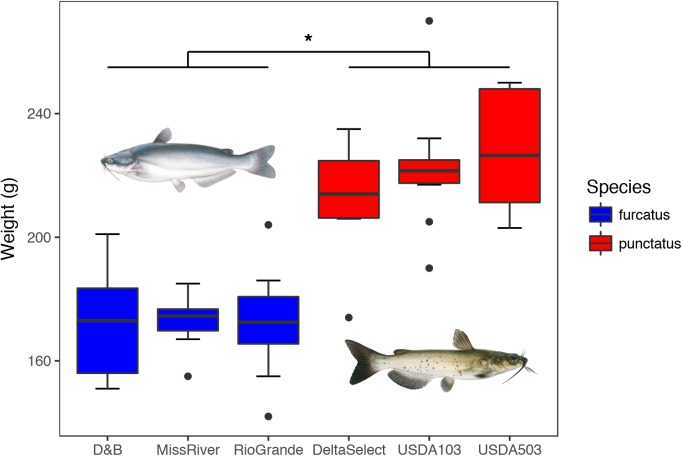
Weights (grams) of individuals sampled within each strain (*n* = 10) at 193 dph, with strains nested by species. A nested one-way ANOVA detected a significant difference between the two species as a whole (*P* ≤ 0.05) as displayed by the asterisk (^∗^), with the three strains of blue catfish *I. furcatus* showing significantly slower growth than that of the three strains of channel catfish *I. punctatus*. No differences were detected at the nested level of fish strain. Images © Joseph R. Tomelleri.

Fish weight indicated that the three strains of blue catfish (172.6 ± 14.35 g; mean ± *SD*) grew significantly slower than that of the three strains of channel catfish (221.43 ± 19.26 g) up to 193 dph, but no intraspecies differences in fish size were detected (**Figure 2**). Fish remained healthy throughout the study and showed no visual signs of disease. After applying the filtering steps outlined in the section “Materials and Methods,” a total of 2,735,946 high-quality 16S rRNA V4 gene amplicon sequences were obtained from the 42 gut-associated microbiota samples, yet a high level of variation in library size was still present after filtering (66,993 ± 43,547; mean ± *SD*). The three environment-associated samples (water and diet) contributed another 278,734 amplicon sequences to the dataset, after filtering. A total of 290 unique bacterial taxa were identified from the gut-associated microbiota samples, with an additional 266 unique taxa found in the environment-associated samples alone.

### Microbiota Diversity Analysis

**FIGURE 3:**
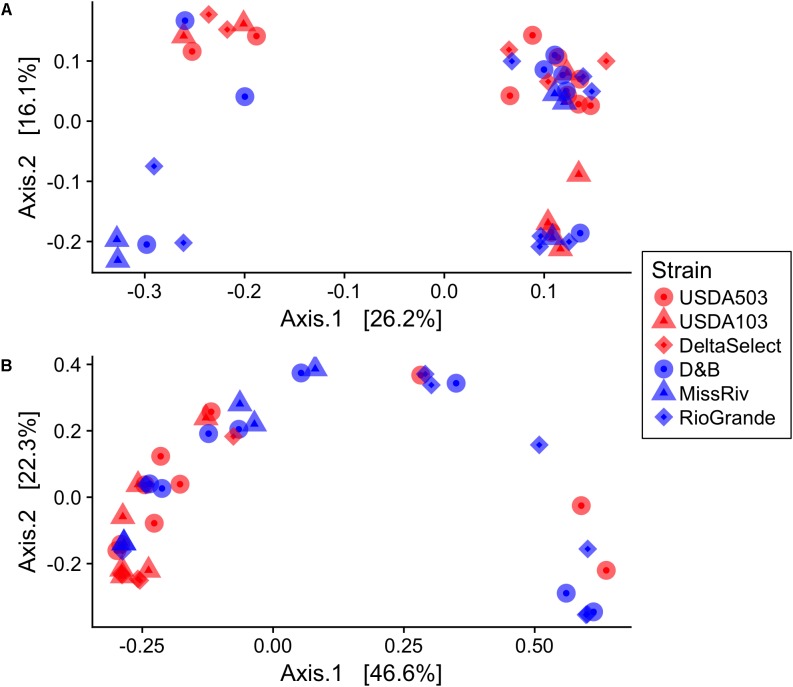
Principal coordinates analysis (PCoA) plots of the gut microbiota beta-diversity of blue catfish *I. furcatus* (blue) and channel catfish *I. punctatus* (red) using **(A)** unweighted UniFrac **(B)** weighted UniFrac. Shapes correspond to the specific fish strains nested within the two ictalurid species. Data were rarefied at the minimum library size (11,631 SVs) prior to beta-diversity analysis.

**FIGURE 4:**
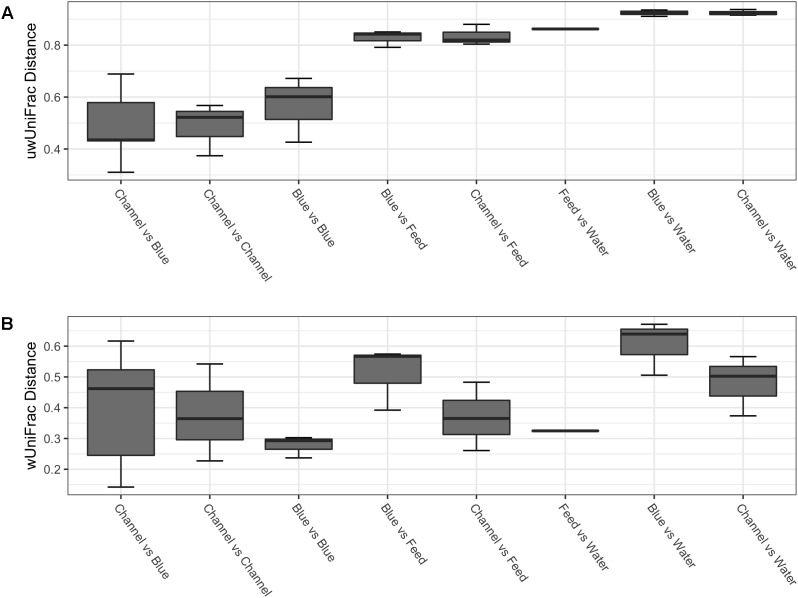
Box plot of beta-diversity distances within and between the channel *Ictalurus punctatus* and blue catfish *I. furcatus* gut-associated microbiota and the environment-associated microbiota (feed and water) samples included in the study using **(A)** unweighted UniFrac **(B)** weighted UniFrac distances. Data were rarefied at the minimum library size (11,631 SVs) prior to beta-diversity analysis.

In this study, the alpha-diversity (observed and Chao1) was relatively low for the gut-associated samples (**Table 1**), and no significant differences in alpha-diversity were detected by nested ANOVA across fish species or strain (**Table 2**). Significant differences were, however, detected by Wilcoxon rank-sum tests comparing the observed richness and Chao1 estimates of the environment-associated samples (water and diet) to the gut-associated samples (**Table 2**). In terms of beta-diversity, the permutational test of multivariate dispersion (PERMDISP2) at the level of fish species revealed a significant difference between blue and channel catfish, with the blue catfish gut-associated samples showing slightly greater intra-species variability using weighted UniFrac (*P* = 0.04; Supplementary Figure S1B). No such differences were detected using unweighted UniFrac (**Table 2**; Supplementary Figure S1A). The nested PERMANOVA showed no differences between fish species or strains (**Table 2**). Principle coordinate analysis (PCoA) of beta-diversity shows some separation among the overall bacterial communities detected in gut-associated samples; however, within species and strain variation was greater than interspecies differences (**Figure 3**). As expected, unweighted UniFrac distances show the gut-associated samples both within and between species to be more similar in composition to one another than to that of the environment-associated (diet and water) samples (**Figure 4A**).

**Table 1:** Summary of alpha-diversity results (observed and Chao1 richness estimates) for microbiota samples collected from the gut of channel *Ictalurus punctatus* and blue catfish *I. furcatus* and their environment.

Sample		Observed	Chao1
category	Group	(mean ± *SD*)	(mean ± *SD*)
*I. punctatus*	USDA103	52.50 ± 27.13	71.72 ± 38.05
	USDA503	70.30 ± 24.42	95.67 ± 32.09
	DeltaSelect	49.00 ± 28.57	113.28 ± 106.17
	Category total	60.14 ± 26.75	93.02 ± 57.64
*I. furcatus*	D&B	73.50 ± 19.77	109.37 ± 37.50
	RioGrande	49.50 ± 18.92	59.91 ± 22.83
	MissRiv	57.80 ± 20.10	102.9 ± 39.80
	Category total	60.62 ± 21.47	88.91 ± 39.27
Environmental	Feed	171^A^	188^A^
	Water	197.50 ± 7.78	221.50 ± 2.12
	Category total	188.67 ± 16.26	210.33 ± 19.40


*Results shown were calculated after removal of non-functional taxonomy (cyanobacteria/chloroplast, NA, or unknown phylum), samples with less than 10,000 assigned reads, and SVs accounting for less than 1e^-*5*^ total relative abundance. ^*A*^Group consists of only one sample; no standard deviation displayed.*

**Table 2:** Summary of statistical tests conducted on the microbiota samples collected from the gut of channel *Ictalurus punctatus* and blue catfish *I. furcatus* and their environment (water and diet).

Parameter	Statistical test	Index	Main factor *P*-value	Nested factor *P*-value
Alpha-diversity (Gut vs. Environ.)	Wilcoxon rank-sum test^A^	Observed	0.004^∗^	
		Chao1	0.007^∗^	
Alpha-diversity	Nested ANOVA	Observed	0.947	0.108
		Chao1	0.939	0.068
Beta-diversity	PERMDISP2	UniFrac	0.981	
		wUniFrac	0.040^∗^	
	Nested PERMANOVA	UniFrac	0.185	0.875
		wUniFrac	0.211	0.095
Beta-diversity vs. host genetic distance (Rst)	Mantel test	UniFrac	0.806	
		wUniFrac	0.333	


*^*A*^Statistical test conducted between sample type (gut-associated vs. environment-associated), all other tests conducted using only gut-associated samples tested by the grouping factors of fish species (main) and fish strain (nested). ^∗^Indicates a rejection of the null hypothesis of no difference among experimental groups (*P* ≤ 0.05).*

### Microbiota Composition and Potential Function

**FIGURE 5:**
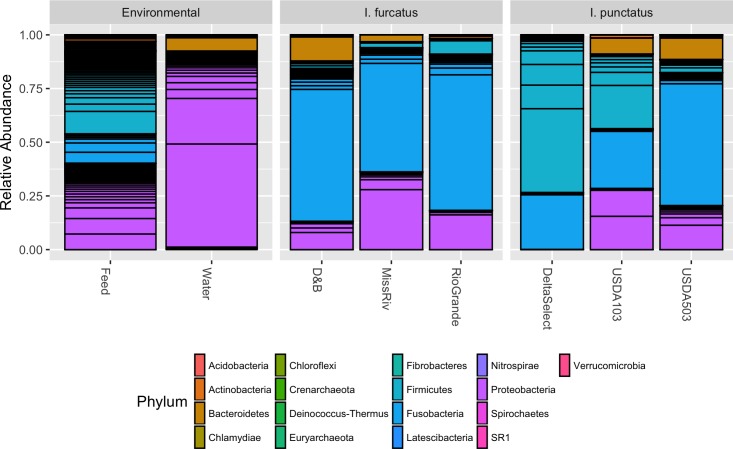
Phylum level RDP v14 taxonomy applied to the 16S rRNA V4 gene amplicons detected in environment-associated and gut-associated microbiota samples taken from strains of blue *I. furcatus* (*n* = 21) and channel catfish *I. punctatus* (*n* = 21) at 193 days post hatch. Reads assigned to cyanobacteria/chloroplast, NA, or unknown at the phylum level, samples with less than 10,000 processed reads, and SVs accounting for less than 1e^-5^ of the relative abundance were removed prior to plotting. Results are displayed as mean relative abundance for each fish strain, with horizontal black lines delineating the abundance of unique SVs assigned within a single phylum.

**FIGURE 6:**
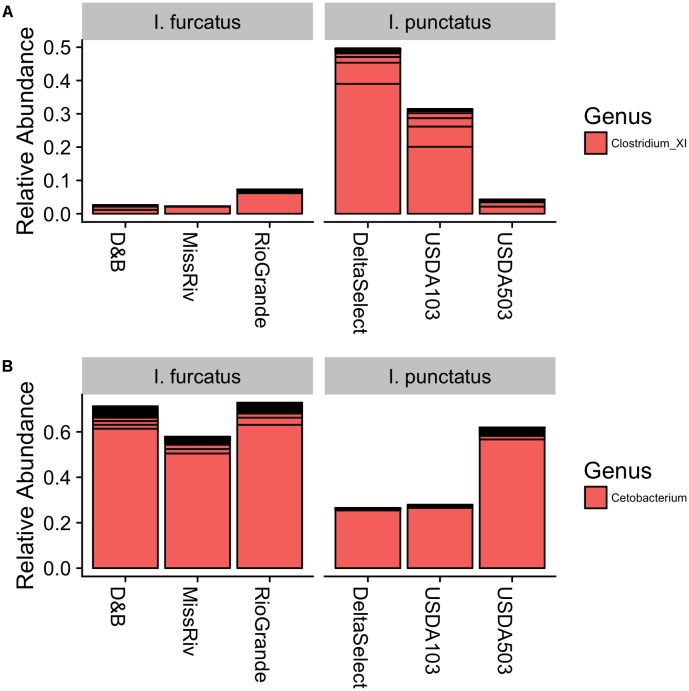
Relative abundance of the differentially abundant microbiota, **(A)** *Clostridium* and **(B)** *Cetobacterium*, detected by ANCOM between blue *Ictalurus furcatus* and channel catfish *I. punctatus*. Results are displayed as mean relative abundance for each fish strain, with horizontal black lines delineating the abundance of unique SVs assigned within a given genus.

The dominant bacterial phyla detected within the gut-associated microbiota of the ictalurid catfish in this study were Fusobacteria, Proteobacteria, Firmicutes, and Bacteroidetes, with other less abundant phyla detected as well (**Figure 5**). Bacteria from the phylum Fusobacteria dominated the microbiota of each strain of blue catfish (D&B 71.2%, Rio Grande 72.8%, Miss River 57.9%), as well as the USDA503 channel catfish (61.9%), while bacteria from the phylum Firmicutes accounted for the greatest relative abundance in the Delta Select and USDA103 channel catfish strains (73.4 and 34.7%, respectively). Interestingly, Fusobacteria were much less abundant within the diet and water microbiota samples (13.6 and >0.001%, respectively). Firmicutes, however, were detected at rather high levels in the diet-associated microbiota sample (38.6%; **Figure 5**), potentially explaining the results found in the gut microbiota samples from the Delta Select and USDA103 strain individuals. This is further supported by the reduced beta-diversity distance between the channel catfish gut samples and the feed sample using weighted UniFrac (**Figure 4B**). The most abundant bacterial phylum detected in the environment-associated microbiota was Proteobacteria, accounting for 40.3 and 90.2% of the diet and water microbiota, respectively. All phyla that were detected in the gut-associated microbiota samples were also present in the environment-associated samples, however, multiple phyla were detected in the environment-associated samples that were not present in the gut-associated samples. Taxonomic data at the genus level for the gut microbiota are shown in Supplementary Figure S2. When conducting differential abundance analysis between the two ictalurid fish species, ANCOM detected two differentially abundant bacterial genera (**Figure 6**). Firmicutes bacteria within the genus Clostridium XI were shown to be consistently more abundant in channel catfish individuals (**Figure 6A**), while Cetobacterium within the phylum Fusobacteria were found to be more abundant in blue catfish (**Figure 6B**).

**FIGURE 7:**
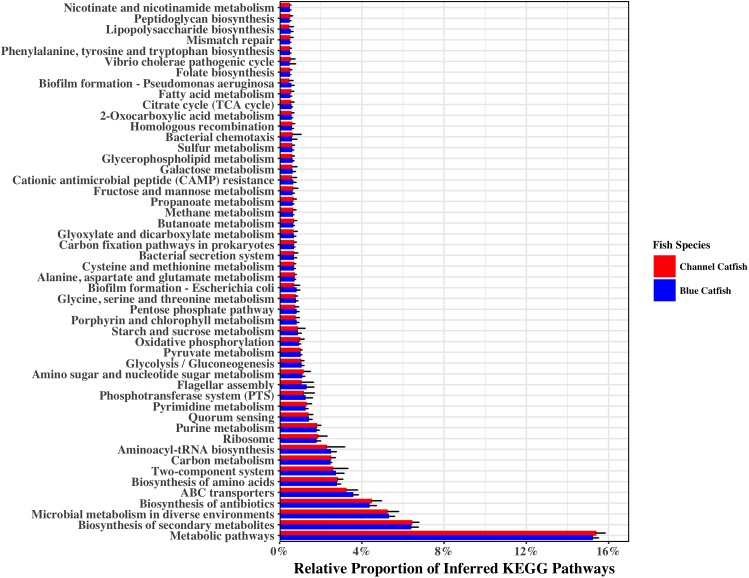
Relative proportion of the 50 most abundant piphillin inferred KEGG pathways associated with the gut microbiota of channel catfish and blue catfish. No statistically differences (FDR ≤ 0.05) in pathway abundances among the fish species were detected for the pathways shown.

The metagenomic functions inferred by piphillin to be associated with the gut microbiota detected in this study showed the predominant bacterial pathway features to be related to microbial metabolism, nutrient processing, antimicrobial biosynthesis, and to a lesser extent pathogenicity (**Figure 7**). Of the 288 inferred metagenomic KEGG pathway features, only two were found to be differentially abundant between the two fish species by DESeq2 (FDR ≤ 0.05), with one feature representing the biosynthetic pathway for beta-lactam antibiotics and the other feature being associated with the potential pathogenicity of *Vibrio* bacteria (Supplementary Table S3). Both of these differential pathway features were found to be of greater abundance in channel catfish, although both features were only minor components of the total inferred metagenomic contents. Most inferred pathway features were of similar abundance between the two fish species (**Figure 7**).

### Host Genetics and the Relationship With Microbiota

**FIGURE 8:**
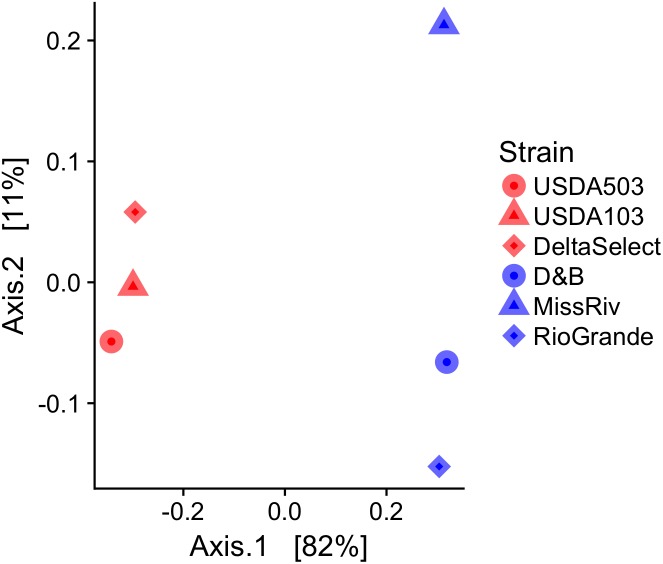
Principal coordinates analysis (PCoA) plot of mean pairwise host genetic distance (Rst) among strains of blue catfish *Ictalurus furcatus* (blue) and channel catfish *I. punctatus* (red). Genetic distances were determined using AMOVA-based Rst calculations using codominant genotypes from individuals within each strain (*n* = 20) across 20 microsatellite loci.

To quantitatively measure the relationship among the host genetics of each strain of ictalurid catfish in this study, microsatellite genotype data from across the host genome were collected and analyzed for pairwise genetic distance (Rst). To reduce the effect of PCR artifacts such as null alleles, individual samples were required to have ≥85% valid genotype results (non-zeros) across the 22 microsatellite loci, and 2 loci were removed from the dataset because ≤90% of the total samples contained valid genotypes at those loci. Filtering yielded genotypic data across 20 microsatellite loci for 20 individuals from each strain. Population level observed and expected heterozygosity across the 20 included microsatellite loci can be seen in Supplementary Figure S3. Plotting of the mean pairwise genetic distance (Rst estimates) among the six fish strains evaluated in this study using PCoA ordinations showed clear separation between the strains within the two ictalurid species (0.657 ± 0.023; mean ± *SD*) (**Figure 8**). Additionally, genetic distances among the three domestic strains of channel catfish (0.179 ± 0.135; mean ± *SD*) were less than that detected among the three domestic strains of blue catfish (0.279 ± 0.106; mean ± *SD*) (**Figure 8**).

**FIGURE 9:**
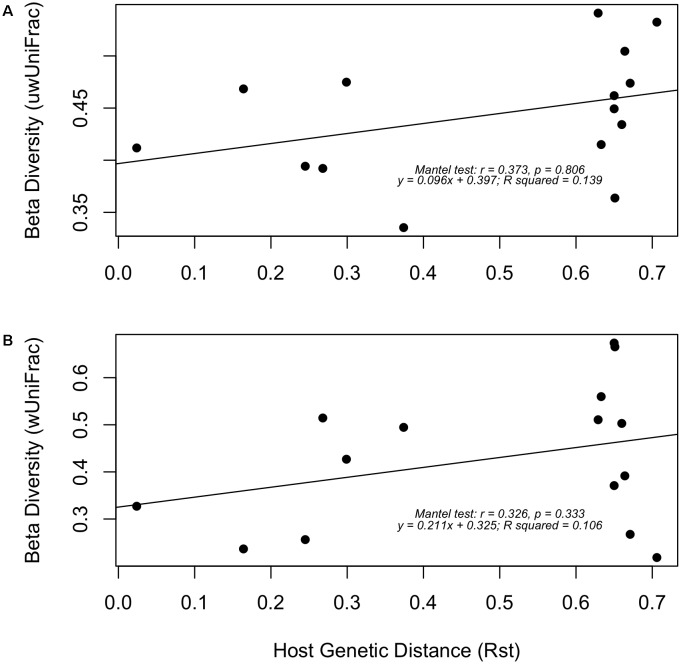
Linear regressions between mean pairwise host genetic distances (Rst) and mean gut microbiota beta-diversity using **(A)** unweighted UniFrac and **(B)** weighted UniFrac distances. Mantel test statistics and regression coefficients are listed within each plot. No significant correlation observed.

Linear regression analysis and Mantel tests were used to evaluate the relationship among mean pairwise host genetic distance (Rst) and mean pairwise gut microbiota beta-diversity (weighted and unweighted UniFrac) in the six strains of ictalurid catfish (**Figure 9**). No significant correlations between host Rst genetic distance estimates and gut microbial beta-diversity were detected using either test or beta-diversity metric.

## Discussion

Many studies on teleost microbiota have suggested that host-genotype best explains the separation in gut-associated microbiota assemblages among various species and even families of finfish. In their characterization of eight different species of freshwater fish, Li J. et al. (2014) found significant correlations among gut bacterial composition and host phylogeny based on cytochrome b sequence analysis. Further, Navarrete et al. (2012) observed that family-wise genetics was capable of overcoming stark differences in dietary composition in explaining gut-associated microbiota composition. However, it is also common for researchers studying the gut microbiota of finfish to find or suggest that environment is among the strongest factors influencing microbiota compositions. Dehler et al. (2017) observed significant separation in the gut-associated microbiota of Atlantic salmon *Salmo salar* that were derived from the same hatchery but raised in separate environments for 8 months. Additionally, Giatsis et al. (2014) demonstrated that various rearing systems could produce significant separation in the gut-associated microbiota composition of larval tilapia *Oreochromis niloticus* cohorts. The contradictory results of these studies suggest environmental forces can often be overwhelmed by host genetic differences with regard to microbiota composition. At other times, environment appears capable of driving strong differentiation in the microbiota assemblages among individuals with common life history and genetics. As the first study to characterize the gut-associated microbiota of multiple strains and species of ictalurid catfish, the present study is meant to further dissect the role of both environment and host genetics on the gut-associated microbiota of aquacultured teleosts by rearing genetically distinct hosts within a common environment under equivalent husbandry practices. The results of our study suggest that a shared environment throughout life can indeed overcome robust differences in host genetics toward shaping the bacterial assemblage of gut-associated microbiota in the commercially and scientifically important ictalurid congeners included in our experiment.

The effects of shared environment and husbandry-practices on the microbiota seen in this study occurred primarily through indirect effects on the microbial ecology and not directly through microbe transfer from the environment, as is suggested by the stark difference in host-associated and environment-associated microbiota (**Table 2** and **Figure 5**). It is likely that the shared water chemistry, husbandry practices, and nutrient inputs administered throughout this study acted together to indirectly shape the gut bacterial communities. These common selective forces appear to have moderated any differences in microbiota composition that may have been present due to factors shaped by host genetics. Previous studies have shown environmental parameters such as water salinity to be a strong influencer of microbial ecology (Lozupone and Knight, 2007; Sullam et al., 2012), and it is likely that other aquatic environmental parameters also have strong influences on the assemblage of gut-associated microbiota. While many studies hypothesize or show that environmental parameters are exerting a selective force upon the gut microbiota assemblages in fish, these studies, including the present research, rarely quantify or attempt to manipulate the environmental parameters. For pre- and probiotics to become useful and effective in the aquaculture industry, it is imperative that future studies further evaluate the role that various environmental parameters (salinity, temperature, pH, oxygen saturation, hardness, conductivity, nitrogen cycling, etc.) might have on shaping the fish gut microbiota. Additionally, research on fish microbiota has already shown that the dietary inputs of nutrients and microbes (Ingerslev et al., 2014; Smith et al., 2015) serve as a strong influencer of microbiota assemblages. However, a better understanding of the effects of particular dietary nutrients on the ecology of gut microbiota, as well as dietary inoculation mechanisms, is still required to fully understand the role that environment plays in influencing fish microbiota compositions.

When considering the factors affecting the colonization dynamics of fish, dispersion of gut-associated microbiota between hosts should also be considered, as the aquatic environment in which fish live serves as an ideal medium for transferring or inoculating microbes (De Schryver and Vadstein, 2014). The dispersion of microbiota between hosts can in turn diminish differences among hosts and result in gut-associated microbiota more closely matching the environment-associated assemblages. Recently, Burns et al. (2017) used an insightful study design to demonstrate that inter-host dispersal of microbiota can mitigate even exaggerated genotypic differences among hosts by comparing the microbiota of either co-housed or solitarily reared wild-type zebrafish *Danio rerio* and immune-compromised zebrafish with a CRISPR/Cas9 myd88 gene knockout, a key gene in the toll-like receptor pathway involved in regulating host–microbiota interaction. Results from that study suggest that the dispersal of microbiota between hosts in an aquatic environment can indeed overcome host genotypic differences. However, in the current study, the constant flow-through environment in which each fish strain was reared, with its rapid water turnover rate, should have greatly reduced the effects of within tank microbial dispersal. In addition, while all fish received the same diets and inflowing water throughout life, individuals within each strain in the present study were cohoused, separate from individuals of other strains. Despite this, only minor differences were detected when comparing the gut-associated microbiota of individuals using the nested statistical design (fish strains with fish species).

Overall, the gut-associated microbiota composition detected among the ictalurid catfish in this study agrees with the results of the limited studies conducted on channel catfish. In a study that characterized the gut-associated microbiota of three commercially important freshwater species collected from a farm-pond, Larsen et al. (2014) observed channel catfish gut-associated microbiota to be dominated (94.02%) by *Cetobacterium somerae*, which was also detected as a dominant microbe in the present study (**Figure 6B** and Supplementary Figure S2). However, Larsen et al. (2014) observed microbes from the genus *Clostridium* to be present at rather low levels (0.3–0.2%), contrary to the results found in the channel catfish within our study (**Figure 6A** and Supplementary Figure S2). These microbes, *Cetobacterium* and *Clostridium XI*, were the most dominant bacteria detected and also the only microbes to be determined as differentially abundant between the two catfish species in this study. Additionally, these two bacteria were also present in roughly the same proportion in research conducted in parallel to the present study, in which our group tracked the gut-associated microbiota of a single family of Delta Select channel catfish across developmental stages (Bledsoe et al., 2016). Interestingly, some of the anaerobic microbes from the family Clostridiaceae have been shown to make up a substantial portion of the human gut-associated microbiota, where they appear to serve a mutualistic or beneficial role to their host by helping to maintain proper membrane integrity and permeability and also provide metabolic and pathogen exclusion functions (Lopetuso et al., 2013; Galperin et al., 2016). As a common anaerobic inhabitant of the freshwater fish gut, *Cetobacterium* is a genus of bacteria known to be involved in vitamin metabolism and the production of antimicrobial peptides (Tsuchiya et al., 2008; Roeselers et al., 2011).

The differential abundance analysis of bacterial taxonomic composition, as conducted by ANCOM in this study, detected only two differentially abundant bacteria, *Cetobacterium* and *Clostridium X* (**Figure 6**). The ANCOM test, which utilizes log-ratio tests to account for the compositional nature of microbiota data (Mandal et al., 2015), was chosen to maintain statistical power while avoiding the inflated false-discovery rate that can accompany other techniques when analyzing data with varying library sizes (Weiss et al., 2017), as was present in this study. Differential abundance testing of the inferred KEGG Pathways by DESeq2, supported the results of ANCOM, with only two functional features being detected as differential between the blue and channel catfish gut bacterial communities (**Figure 9** and Supplementary Table S3). Overall, the pathway features inferred by piphillin are aligned with the reported functions of teleost microbiota, including involvement in nutrient metabolism, antimicrobial/antibiotic biosynthesis, and potential pathogenicity. However, it should be noted that the utility of piphillin and other similar metagenomic inference tools relies heavily on the quality and abundance of reference genomes included in the algorithm and therefore such tools tend to perform better on samples taken from highly studied environments such as human-associated microbial communities (Iwai et al., 2016).

The host genotyping conducted within this study showed that despite the lack of significant differences in the gut microbiota (**Table 2** and **Figure 3**) significant genetic differences were present in the strains and species of catfish (**Figure 8**). While a modest number of individuals from each strain were genotyped (*n* = 20), previous research has shown that roughly 25 individuals are sufficient to capture population level allele frequencies for reliable estimation of genetic distances between populations (Hale et al., 2012). Four out of the six strains utilized in this study experienced strong selective pressures, with the exception of the Mississippi River and Rio Grande River blue catfish strains, and all strains have experienced domestication pressures. Thus, the slight differences found between observed and expected heterozygosity among the microsatellite markers were expected (Supplementary Figure S3). The three strains of channel catfish which are closely related and have received similar selection pressures showed much less separation of host genetics than did the blue catfish, which are much less closely related and represent more unselected genotypes (**Figure 8**).

Despite the lack of significant correlations in this study between host genetics and microbiota composition, the slightly positive slope of the linear regressions and the moderate R values of the Mantel tests using either UniFrac metric (**Figure 9**) might suggest that the greater the genetic distance between populations the more likely those populations are to have disparity in their gut microbial communities. In a study comparing the gut microbiota of multiple wild populations of threespine stickleback *Gasterosteus aculeatus* with varying levels of geographic and genetic separation, Smith et al. (2015) found a significant Mantel correlation (*R* = 0.651, *P* = 0.02) among host genetic distance (Rst) and gut microbial beta-diversity (unweighted UniFrac). Similarly, Li J. et al. (2014) showed a significant correlation among host-genetics and gut-associated microbiota assemblages in eight different freshwater fish species. Although it should be noted that potentially confounding covariates of host genetics are also present in those studies, including differences in dietary inputs, geographical location (environment), and life history. This likely explains the stronger correlations found in those studies, compared to the present research, in which habitat, husbandry, and diet were all held constant among the experimental individuals.

To summarize, our study is the first comparison of the structure and potential function of the gut microbial communities of multiple strains of the commercially and scientifically important blue and channel catfish. Findings show that despite rather clear distinctions in genome-wide microsatellite-based genetic distance, there were no significant differences in gut-associated microbiota composition among strains and species of these ictalurid catfish. Our results show that the environment-associated (diet and water) microbiota is starkly different than that of the fish gut-associated samples. However, shared environment, diet, and husbandry practices appeared to moderate the microbiota composition associated with hosts of divergent genetic backgrounds. This suggests that environment may have an indirect yet strong influence on shaping the bacterial communities of teleost fish. While previous studies have shown strong correlations among host-genotype and gut microbiota beta-diversity, the present study suggests such results may be have been influenced by other uncontrolled co-variates such as geographical location (i.e., environmental parameters) and dietary inputs. Results from this study have implications on future basic research on teleost microbiota assemblage dynamics as well as on applied catfish (*Ictaluridae*) research, demonstrating that a common environment and life history can diminish genetic-based differences in gut microbiota composition and function.

## Author Contributions

JB, BP, KS, and BS conceptualized and designed this work. JB and GW were involved in sample processing and data collection. JB conducted the primary investigation, bioinformatic and statistical analysis of the data, and wrote the manuscript. KS, BP, GW, and BS provided resources and analysis tools. KS, BP, GW, and BS reviewed and edited the manuscript. BP and BS were responsible for funding acquisition. All authors approved the final manuscript.

## Conflict of Interest Statement

The authors declare that the research was conducted in the absence of any commercial or financial relationships that could be construed as a potential conflict of interest.
