# Supplementary material for: Comparison of Channel Catfish and Blue Catfish Gut Microbiota Assemblages Shows Minimal Effects of Host Genetics on Microbial Structure and Inferred Function
Source: Front Microbiol. 2018 May 23;9:1073. doi: 10.3389/fmicb.2018.01073 (PMC5974930; doi:10.3389/fmicb.2018.01073)
Supplement: Supplementary file 1 [file Presentation_1.pdf]

Comparison of channel catfish and blue catfish gut microbiota assemblages shows minimal effects of host genetics on microbial structure and inferred function

Jacob W. Bledsoe*, Geoffrey C. Waldbieser, Kelly S. Swanson, Brian C. Peterson, Brian C. Small

[*] Corresponding Author: bledsoe@uidaho.edu

Supplementary Figures and Tables

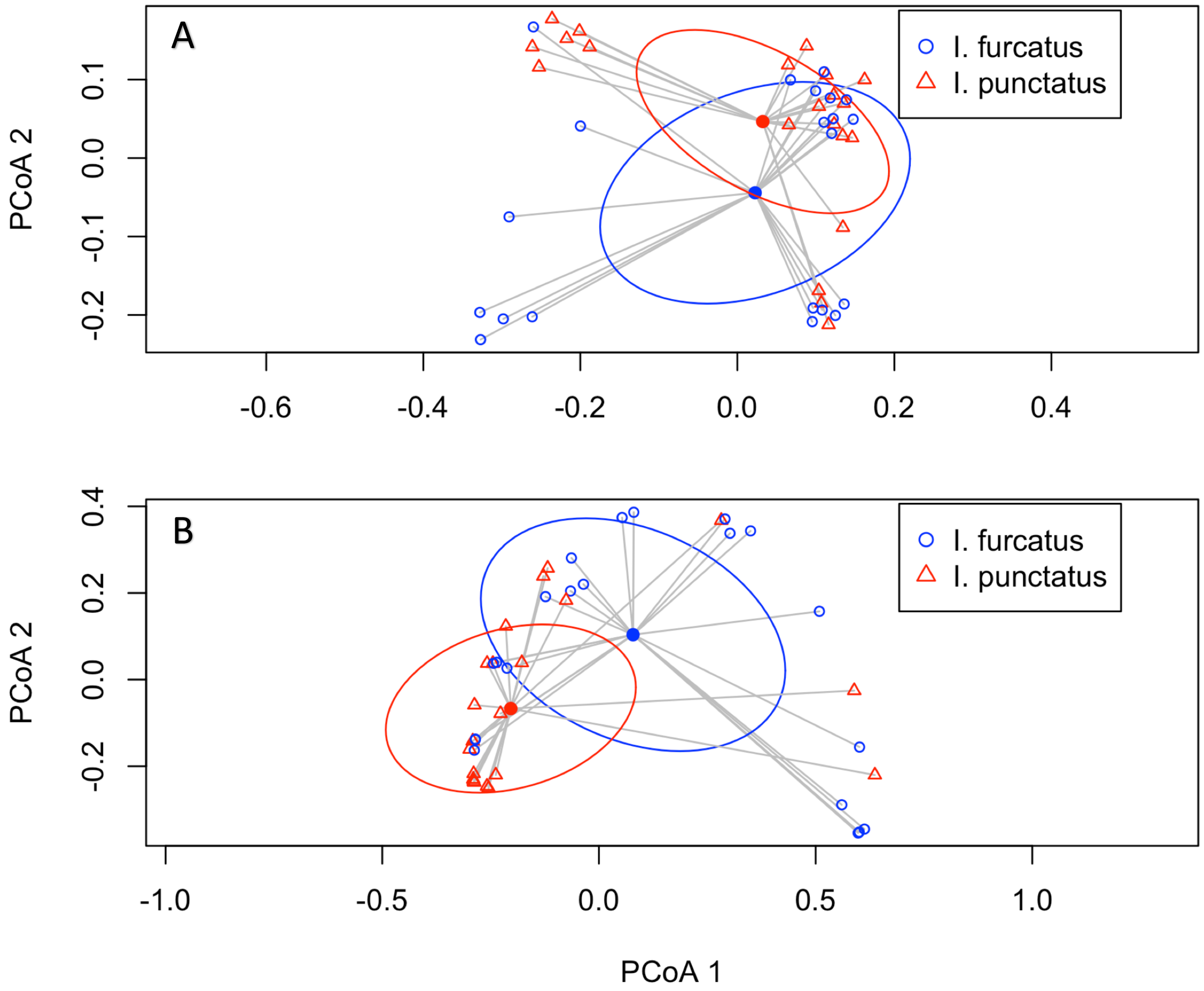

Supplemental Figure S1. Principal coordinates analysis (PCoA) plots showing the multivariate dispersion of (**A)** unweighted UniFrac and (**B**) weighted UniFrac beta diversity of the gut microbiota of blue catfish *I. furcatus* (blue circles; n = 21) and channel catfish *I. punctatus* (red triangles; n = 21). Filled circles represent the group centroids and ellipses represent 1 standard deviation of multivariate dispersion from the centroid.

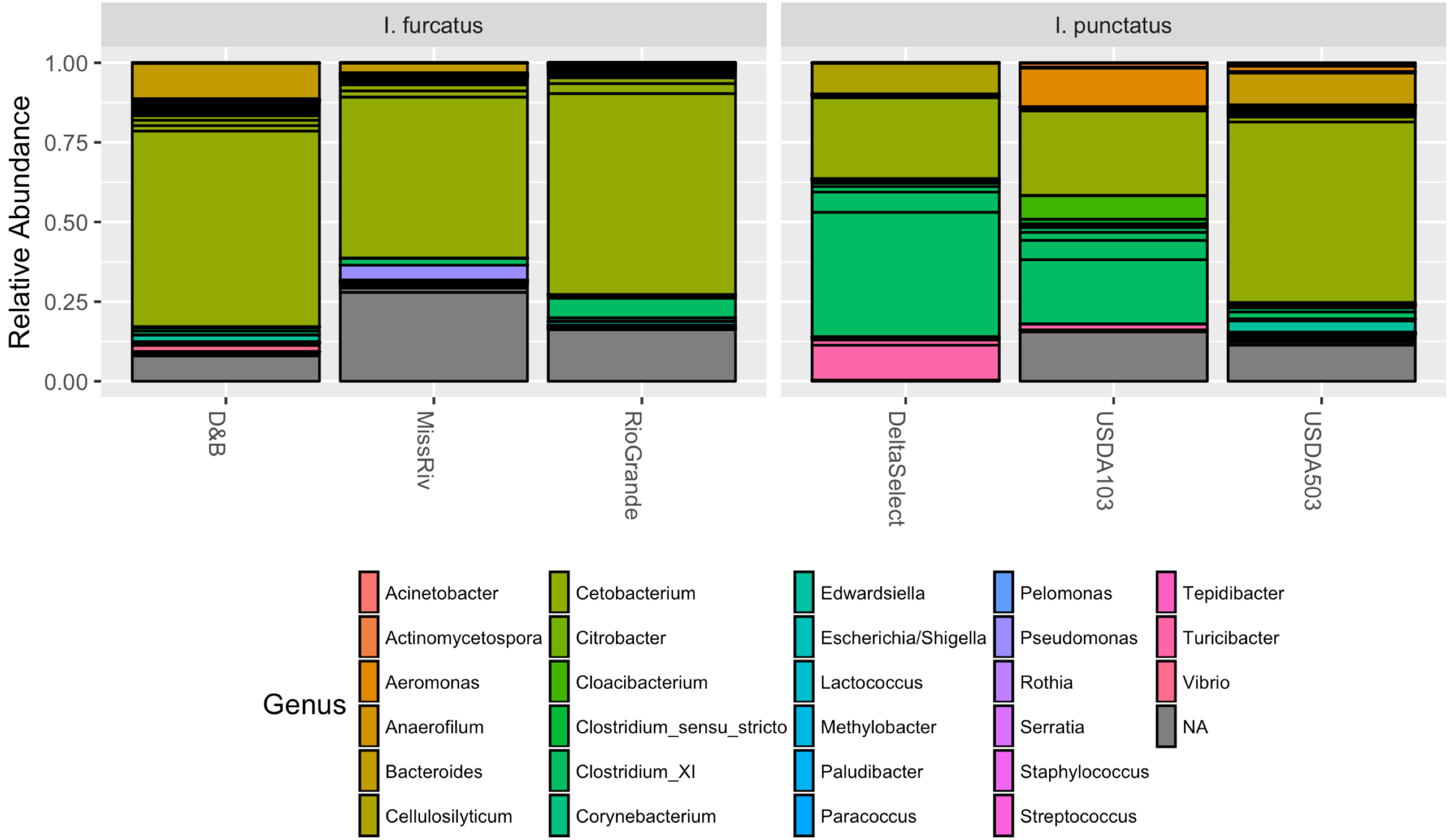

Supplemental Figure S2. Genus level RDP v14 taxonomy applied to the 16S rRNA V4 gene amplicons detected in gut-associated microbiota samples taken from strains of blue *I. furcatus* (n = 21) and channel catfish *I. punctatus* (n = 21) at 193 days post hatch. Reads assigned to Cyanobacteria/Chloroplast, NA, or unknown at the phylum level, samples with less than 10,000 processed reads, and sequence variants accounting for less than 1e^{-5} of the relative abundance were removed prior to plotting. Results are displayed as mean relative abundance for each fish strain, with horizontal black lines delineating the abundance of unique 16S rRNA V4 gene sequence variants assigned within a single genus. Only the top 200 most abundant sequence variants are included.

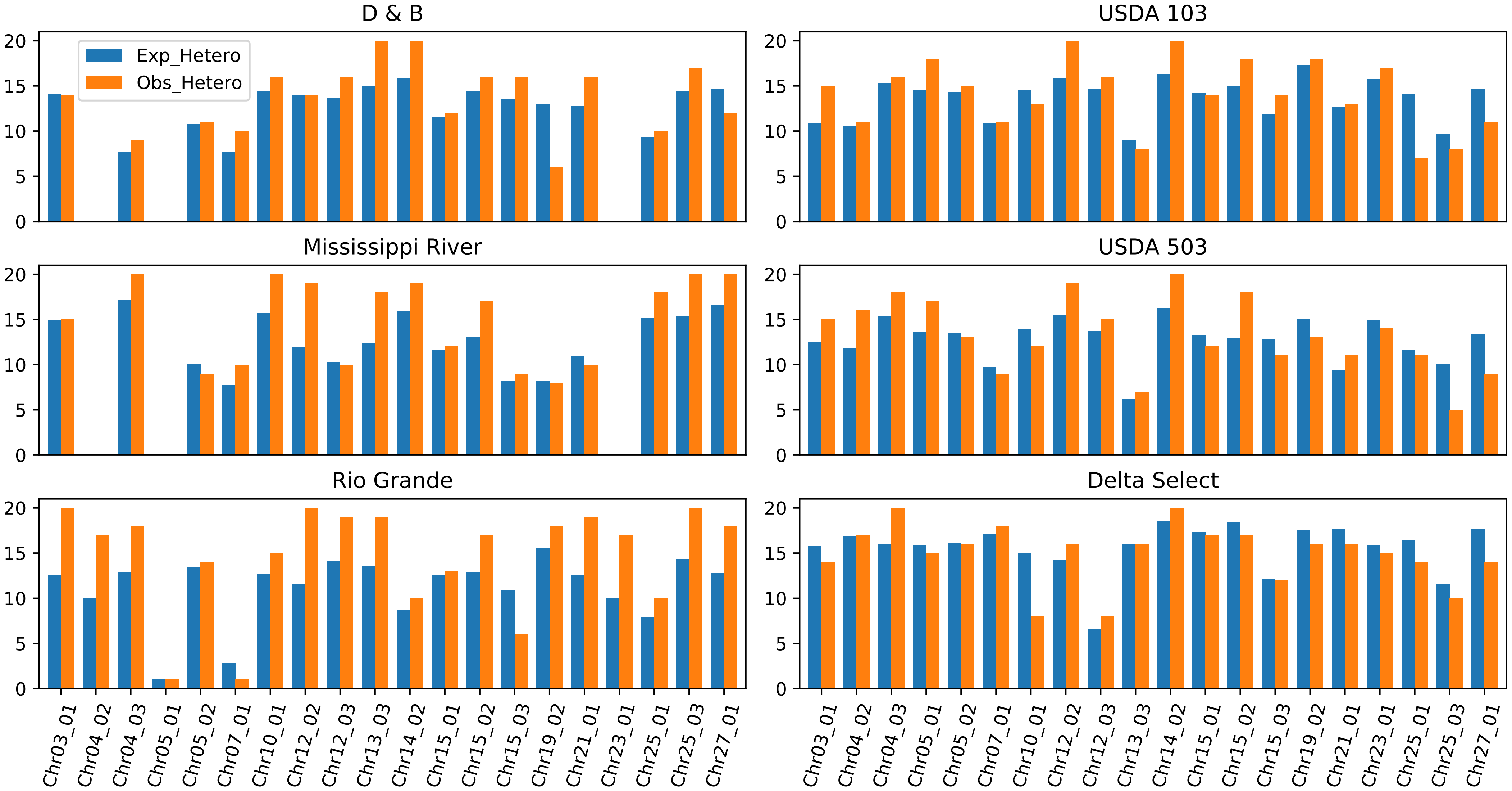

Supplemental Figure S3. Observed (orange) and expected (blue) heterozygosity for each fish strain (n = 20) across the 20 microsatellite loci included in the study. Expected values represent the heterozygosity under Hardy-Weinberg equilibrium. Left panels are strains of blue catfish *I. furcatus* and right panels are strains of channel catfish *I. punctatus*. PCR primers and repeat motifs used in this analysis can be found in Supplemental Table 2.

Supplemental Table S1. Proximate analysis of commercial diet being fed at the time of sampling.

Diet	Pellet Size (mm)	Dry Matter (%)	Crude Protein (%)	Crude Fat (%)	Ash (%)
MiniPellet[A]	2.4	90.7	34.4	4.5	8

[A] Fishbelt Feeds Inc., MS, US. – Diet is a lost-cost formulation with varied ingredient formulations from batch to batch.

Supplemental Table S2. Locus specific metadata for multiplexed PCR-sequencing based microsatellite analysis conducted within this study.

Locus	Repeat Motif	Forward Primer (5' - 3')	Reverse Primer (5' - 3')	Position in Reference Genome (*I. punctatus*)
Chr03_01	AC	GTTATTTCTACTACTGGCTACTGTAC	AAGCCTGGAGTATGTGCAGGCT	31,041,248
Chr04_02	AAG	CTTCCTCATACAGCACACAG	CGCGCTTTCATAAACAGGAAGTGGCACTG	30,690,440
Chr04_03	AAT	TAACCTGCTCTAGTCTACAT	GCAAATAAATCAAAACCTGAGT	819,886
Chr05_01	AAAT	ACTCGGTCTTGGGCTTTAG	ATTGGTTTCGAAACCTCATCCGTCTCCGAC	1,833,406
Chr05_02	AAT	AGTGCACATTTTAAAGACTGGC	CACAAGGGCAACTGAGATGGAT	28,637,866
Chr07_01	ATT	TTGTAAGTGACCTTGGTTGTGA	ATTAAATCTAATTTCAGTATGGGC	4,954,699
Chr10_01	AAG	GATTAGTACAACATGAAAACACAAGA	CCGAGATTCATTTGTCGGTCATGTC	18,379,264
Chr12_02	AG	TCAACCTGGTCACTTAACTCTG	AATTAGGATAGAGAGAAATTAAATAGAAGCAAGAT	13,610,928
Chr12_03	AAG	CTACAGTTTGGTCCGTATGATC	GCTAGGGAAAGAAAGTTGGCA	1,882,749
Chr13_03	AAT	GGTAGGAACTGGCCAAAATGG	CCAATGAGGACAACAAATTTTGTGTTCAAGTTG	19,015,384
Chr14_02	AG	AAACAGCCCAAGGCCAAAGG	TTGTTGTCACTCAATTCCTATGTA	24,191,069
Chr15_01	ATAG	ATCATTTATCTGTGACATTTTATGG	TCTCAATTAAGGGCAATTTACAGA	1,070,242
Chr15_02	AAC	ACTGAATGACTAAGGAGAAAAC	AAGCATGATAAAGAAAGAAAAA	22,338,963
Chr15_03	AGT	CAAGAATGGGAAGGGTGTACTTA	ATATTGCACATGCACATATGCA	19,465,810
Chr19_02	ATAG	TACCACCTGAGCCACAGCTG	CAGCAGTTAGTGAAAGCCCAGTT	20,156,330
Chr21_01	ATAG	CGTGTCCACCAAGTCCCTG	GTCCTCAAAGAGGAATTT	6,796,744
Chr23_01	ATGG	GATGTTTCAGTGCATTTACTCTG	GCCAGTAAAAAGACAGCTCATCCTGAGATAG	572,870
Chr25_01	AAT	GGCGGTACTGTCCCTGTATG	CAGAAAATGTATACTCTTTTCCAAT	18,118,630
Chr25_03	AAT	TCCCACTAAACTCCATTGTACC	CATCAGATTATCACCAGGATCA	1,474,793
Chr27_01	AAT	CCAATACTGTGATTTAATTTACAATG	TATTTATTCATTTAATATTTACTTGTGTT	10,417,665
